# Limited-View X-Ray Tomography Combining Attenuation and Compton Scatter Data: Approach and Experimental Results

**DOI:** 10.1109/ACCESS.2019.2953217

**Published:** 2019-11-12

**Authors:** Abdulla Desmal, Jeffrey R. Schubert, Jeffrey Denker, Sherman J. Kisner, Hamideh Rezaee, Aaron Couture, Eric L. Miller, Brian H. Tracey

**Affiliations:** Department of Electrical and Computer EngineeringTufts University 1810 Medford MA 02155 USA; American Science and Engineering, Inc. Billerica MA 01821 USA; High Performance Imaging LLC West Lafayette IN 47906 USA

**Keywords:** Inverse problems, computed tomography, reconstruction algorithms, x-rays

## Abstract

X-ray inspection systems are critical in medical, non-destructive testing, and security applications, with systems typically measuring attenuation along straight-line paths connecting sources and detectors. Computed tomography (CT) systems can provide higher-quality images than single- or dual-view systems, but the need to measure many projections leads to greater system cost and complexity. Typically, off-angle Compton scattered photons are treated as noise during tomographic inversion. We seek to maximize the image quality of limited-view systems by combining attenuation data with measurements of Compton-scattered photons, exploiting the fact that the broken-ray paths followed by scattered photons provide additional geometric sampling of the scene. We describe a single-scatter forward model for Compton-scatter data measured with energy-resolving detectors, and demonstrate a reconstruction algorithm for density that combines both attenuation and scatter measurements. The experimental results suggest that including Compton-scattered data in the reconstruction process can improve image quality for density reconstruction using limited-view systems.

## I. Introduction

X-ray imaging is critical in medical [1], industrial [2] and airport security [3], [4] applications. While medical applications have attracted the greatest attention, security scanning applications such as luggage screening offer several unique challenges. Because a wide variety of materials are encountered in luggage screening, accurate material identification is important, leading to interest in using energy discriminating detectors to improve material identification [5]–[9]. A second key difference is that while CT systems used in medical imaging are generally able to collect data projections at a large number of angles fully encircling the object, access to the object from multiple views is limited in many security applications, including luggage screening and kVp spectral CT [10]. There have been a variety of investigations into limited-view tomography methods, several of which exploit energy-resolved measurements and enforce similarity across energy channels [10]–[13]. However, the limited-view CT problems remains quite challenging, even more so when coupled with the need for accurate material identification.

Our hypothesis is that image quality can be improved in limited-view tomography by processing not only straight-line attenuation projections through the object, but also broken-ray data created through Compton scattering. Scattering occurs when photons travel from the source to a scattering object, deflect via Compton scattering to a new angle, and then are measured at a detector. Including these broken-ray paths in the inversion process dramatically increases the number of geometric ray paths through the investigation domain, and thus holds the potential to reduce image artifacts. In addition, there are indications that Compton scatter data has the potential to improve materials identification as it provides a strong contrast mechanism comparing to total attenuation [14], [15]. Compton scatter tomography has been explored previously and has been shown to have advantages over conventional CT systems in nondestructive evaluation applications [16] and materials characterization [17]. However, processing this scattered data brings several challenges, notably the low number of counts associated with these raypaths and the computational burden of modeling the additional paths (the forward model for Compton scatter tomography models attenuation from the source to the scattering point, the scattering process itself, and attenuation of the scattered photon as it travels to the detector). In addition, the forward model could become computationally intractable in situations where multiple scattering dominates over single-scattering.

Analytic Compton scattering tomography reconstruction methods are available, but are limited to specific data acquisition geometries [18], [19]. Numerical reconstruction algorithms, such as the one discussed in this paper, are applicable to more general geometries. Unlike the work presented here, many previous publications either assume that an X-ray attenuation map is known *a priori* from a traditional CT scan (resulting in a linear mapping from density to observations) [14], or do not fully model the energy dependence of the attenuation [20], [21]. Our forward model is more closely related to that in [22], although that work includes fluorescence effects, which are not important in our application.

This work builds on and extends our recent simulation-based study that combines scattered photons with attenuation data to perform image reconstruction [23]. While that work employed a similar forward model to the one considered here, the inversion approach was entirely different (as a minor note, the current model uses a different solid angle calculation which was proven to better match the experimental results). More specifically, the work in [23] focused on recovering spatial maps of both density and photoelectric coefficients. Because of the smaller problem sizes considered, a Newton type optimization method could be used. An edge preserving regularization method first developed in [24] was used to stabilize the density profile while a nonlocal means regularizer was used for photoelectric. While effective, these regularization methods are computationally intensive, requiring the solution of multiple least-square problems at every “outer” iteration of the Newton method.

Driven largely by the exigencies associated with processing a larger *experimental* data set, the effort here differs significantly from the simulation-based work in [23]. Our goal is to provide an experimental validation of the benefit of combining attenuation and Compton scatter data. Thus, we focus on density inversion, deferring experimental validation of photoelectric coefficient inversion (which is more ill-posed) to future work. Specific contributions of the current work are as follows: a) we develop experimental hardware to support our studies, including design of a novel source collimator, and validate the accuracy of our forward model against experimental data; b) we employ an iterative reconstruction method that scales better to larger-scale problems than the methods from [23]; c) we reduce computational effort algorithmically, by linearizing the attenuation component of our inverse problem using a multi-energy sinogram decomposition method (see Appendix B) that exploits energy-discriminating detectors; and d) we address computational issues by demonstrating an implementation that exploits capabilities of multi-core processors.

The remainder of this paper is organized as follows. We first describe the proposed system geometry and describe the forward models for both Compton-scattered measurements and attenuation data. We then introduce a gradient descent algorithm that combines both attenuation and scatter measurements. In the Results section, we describe an experimental testbed and describe test scenarios. This testbed was used to validate our single-scatter forward model in a recent paper [25], indicating the multiple-scattering effects are negligible for this system. Finally, experimental results are shown for density reconstruction of several objects, demonstrating that incorporating scatter data into the inversion leads to noticeable improvements in image quality and accuracy of density estimates. Finally, we conclude and discuss future work.

## II. Formulation

**FIGURE 1.**
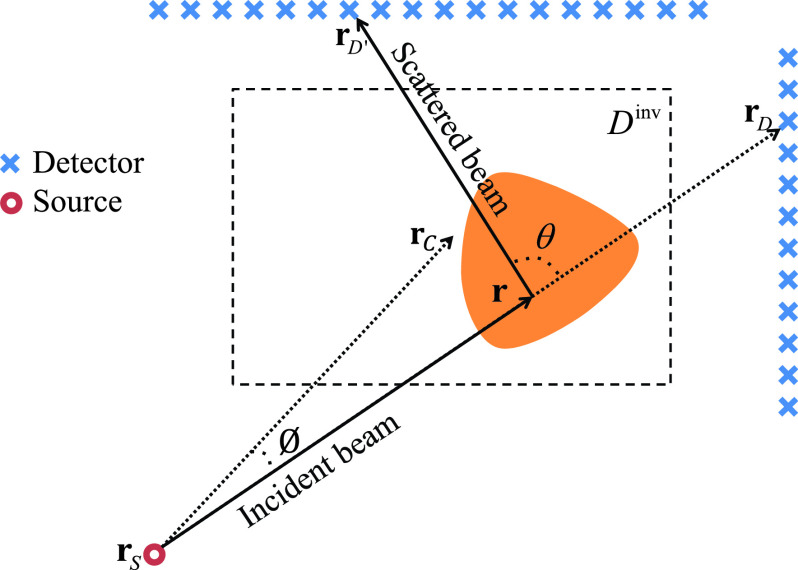
Problem configuration.

In this section, we describe our physical forward model, then outline the reconstruction algorithm. Fig (1) illustrates a two-dimensional cross section of the problem, where the slice plot is over the $x-y$ plane and out-of-plane scattering is neglected (justified experimentally as our measurements are all in-plane). A pencil-beam X-ray source at location $\boldsymbol {r}_{S}$ illuminates the investigation domain $D^{inv}$ at beam angle $\phi $, which is the angle between $\boldsymbol {r}$ and $\boldsymbol {r}_{C}$ that points toward center of $D^{inv}$. During data collection, the angle $\phi $ is rotated to sweep across the domain. The source is moved in discrete angular steps, so in the discussion below, one beam is used to denote data collected for one source at one angle. Some photons travel on a straight-line path to a detector, providing a measurement of attenuation along the path, while others undergo non-coherent Compton scattering and are deflected to other detectors, providing broken-raypath data. For a particular beam, the detector receiving straight-line attenuation data is known as the *primary* detector $D$, while detectors receiving scattered data are known as *secondary* detectors $D'$. Forward models for both types of data are now described.

### A. Forward Model for Compton Scattering

When the incident beam crosses the investigation domain it interacts with material inside, causing non-coherent Compton scattering. Using notation similar to [22], the expected number of Compton-scattered photons collected at secondary detector $\boldsymbol {r}_{D'} $ at an energy $E_{D}'$ is given by:

\begin{align*}&\hspace{-1.8pc}g_{S}(\boldsymbol {r}_{D'},E_{D}')=\int \mathrm I(E_{S})\biggl [\int h(\boldsymbol {r}_{D'},\boldsymbol {r},E')S(\boldsymbol {r},\theta,E_{S}) \\&\qquad \qquad f(\boldsymbol {r},\boldsymbol {r}_{S},E_{S})\delta _{E_{D}'}(E') \delta _{\boldsymbol {r}_{D},\boldsymbol {r}_{S}}(\boldsymbol {r})\rho (\boldsymbol {r}) \mathrm {d}\boldsymbol {r}\biggr]\,\mathrm {d}E_{S}\tag{1}\end{align*}

where $\mathrm I(E_{S})$ is the number of photons emitted by the source at energy $E_{S} $. $f(\boldsymbol {r},\boldsymbol {r}_{S},E_{S})$ computes the attenuation that occurs as the incident photons travel from source location $\boldsymbol {r}_{S} $ to interaction point $\boldsymbol {r} $. Similarly, $h(\boldsymbol {r}_{D'},\boldsymbol {r},E')$ computes the scattered beam attenuation that occurs as the scattered photons travel from interaction point $\boldsymbol {r} $ to secondary detector location $\boldsymbol {r}_{D'} $. Notice that Compton scattering causes an energy shift, so $h(\boldsymbol {r}_{D'},\boldsymbol {r},E')$ depends on the scattered photon energy $E'$. The function $S(\boldsymbol {r},\theta,E_{S})$ computes the fraction of photons scattered toward angle $\theta $ at interaction point $\boldsymbol {r} $ (see Fig. 1). $\rho \left ({\boldsymbol {r} }\right)$ is the material density evaluated at the interaction point $\boldsymbol {r} $. $\delta _{E_{D}}(E')$ is a Dirac delta function that selects for scattered photons with energy $E'$ that matches the energy $E_{D}'$ at the detector. Finally, $\delta _{\boldsymbol {r}_{D},\boldsymbol {r}_{S}}$ is a Dirac delta function defined within $D^{inv}$ over a line connecting $\boldsymbol {r}_{S}$ to the primary detector $\boldsymbol {r}_{D}$.

We now describe the terms in Eq. (1) in more detail. The attenuation functions are computed as:

\begin{align*} f(\boldsymbol {r},\boldsymbol {r}_{S},E_{S})=&\exp \left ({-\int \mathrm \mu (\boldsymbol {r}',E_{S})\delta _{\boldsymbol {r},\boldsymbol {r}_{S}}(\boldsymbol {r}') \mathrm {d}\boldsymbol {r}'}\right)\tag{2}\\ h(\boldsymbol {r}_{D'},\boldsymbol {r},E')=&\Omega _{D'} \exp \left ({-\int \mathrm \mu (\boldsymbol {r}',E')\delta _{\boldsymbol {r}_{D'},\boldsymbol {r}}(\boldsymbol {r}') \mathrm {d}\boldsymbol {r}'}\right)\tag{3}\end{align*}

where $\delta _{\boldsymbol {r},\boldsymbol {r}_{S}}(\boldsymbol {r}')$ and $\delta _{\boldsymbol {r}_{D',\boldsymbol {r}}}(\boldsymbol {r}')$ are Dirac delta functions defined along raypaths that connect $\boldsymbol {r}_{S}$ to $\boldsymbol {r}$ and $\boldsymbol {r}$ to $\boldsymbol {r}_{D'}$, respectively. The solid angle $\Omega _{D'}$ for a small rectangular shape detector is approximated as [26]:

\begin{equation*}\Omega _{D'} \approx {|{\cos ^{-1}\left ({{ {\widehat {\boldsymbol {r}}_{T}}}.{{\widehat {\boldsymbol {r}}_{B}}}}\right)\cos ^{-1}\left ({\widehat {\boldsymbol {r}}_{L}.\widehat {\boldsymbol {r}}_{R}}\right)}|}\end{equation*}

where $\widehat {\boldsymbol {r}}_{T}$, $\widehat {\boldsymbol {r}}_{B}$, $\widehat {\boldsymbol {r}}_{L}$ and $\widehat {\boldsymbol {r}}_{R}$ are unit vectors pointing from the interaction point $\boldsymbol {r}$ to centers of top, bottom, left and right edges of the detector at $\boldsymbol {r}_{D'}$.The attenuation function $\mu \left ({\boldsymbol {r},E}\right)$ in Eqs. (2) and (3) are the attenuation coefficient function, which is formulated in terms of Compton scatter and photoelectric effects as:

\begin{equation*} \mu (\boldsymbol {r},E)=N_{A} \frac {Z(\boldsymbol {r})}{A(\boldsymbol {r})}f_{KN}(E)\rho (\boldsymbol {r})+f_{p}(E)p(\boldsymbol {r})\tag{4}\end{equation*}

where $N_{A} $ is the Avogadro number and $Z(\boldsymbol {r})$ and $A(\boldsymbol {r})$ are the atomic and mass numbers, respectively. $\rho (\boldsymbol {r})$ and $p(\boldsymbol {r})$ are the material density and photoelectric coefficients, while $f_{p} (E)=(E_{0} /E)^{3} $ is the photoelectric energy factor with $E_{0}$ as the referenced energy. An interested reader is referred to [23] for details concerning the computation of the Klein-Nishina cross section $f_{KN}(E)$ in Eq. (4), the scattered energy $E'$ and the scattering factor $S(r,\theta,E_{S})$ in Eq. (1).

Note that Eq. (1) computes the scattered photons that are incident on the detector at energy $E_{D}$, and does not account for the fact that the detector provides an imperfect measurement of photon energy. The actual signal recorded by the detector at energy $E_{q}$ is found by integrating over the detector sensitivity function:

\begin{equation*} g_{D'}(\boldsymbol {r}_{D'},E_{q}) = \int s_{q}(E_{D'}) g_{S}(\boldsymbol {r}_{D'},E_{D'}) dE_{D'}\tag{5}\end{equation*}

where $s_{q}(E)$ is the sensitivity function for the $q^{th}$ energy channel, centered at $E_{q}$. Idealized models for detector sensitivity functions are presented in [27], and model the sensitivity functions as being Gaussians whose bandwidth depends on detector properties. More realistic sensitivity functions can be computed for particular detectors and account for energy-dependence of the sensitivity function; we employ a model for the Multix ME100 detector found in [6].

### B. Discrete Form of the Compton Scattering Model

**FIGURE 2.**
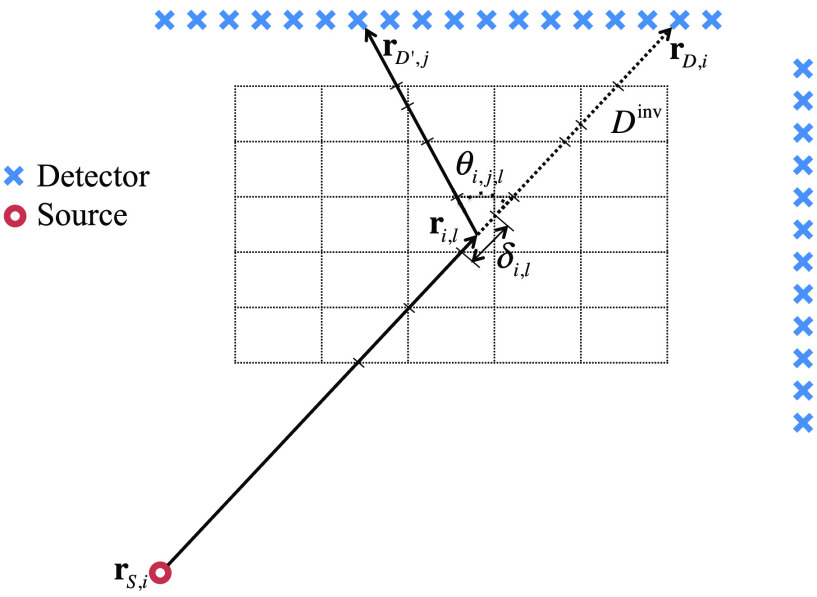
Discretization of the investigation domain $D^{inv}$.

The spatial domain in Eq. 1 is discretized into equal rectangular cells, see Fig. 2, such that the dimension across x and y-axis has $N_{x}$ and $N_{y}$ segments, respectively, and the total number of cells is $N_{Cells}=N_{x} N_{y} $. The source spectrum is discretized into $N_{E}$ energy levels, where the $k^{th}$ level is centered at $E_{S_{k}}$ and has width of $\Delta E_{S}$. Similarly, we discretize the photon energies incident on the detector into $N_{D}$ energy bins, each with bin width $\Delta E_{D}$. Then, the discretized version of Eq. (1) can be written as:

\begin{align*} \boldsymbol {g}_{S}(i,j,m)=&\sum _{k}{} I(E_{S_{k}})\Delta E_{S} \sum _{l}{}h(\boldsymbol {r}_{D',j},\bar {\boldsymbol {r}}_{i,l},E'_{i,j,l,k}) \\&\times \, S(\bar {\boldsymbol {r}}_{i,l,},\theta _{i,j,l},E_{S_{k}})f(\bar {\boldsymbol {r}}_{i,l},\boldsymbol {r}_{S,i},E_{S_{k}}) \\&\times \, \omega (i,j,l,k,m)\delta _{i,l}\rho (\bar {\boldsymbol {r}}_{i,l,})\tag{6}\end{align*}

The indices $i$ and $j$ refer to a particular incident beam and secondary detector in the system, respectively, while the index $m$ indexes the fine scale energy bins into which we aggregate the scattered photons prior to accounting for the finite energy resolution of the detectors in Eq. 10. The index $l$ indicates a discretized cell (that contains the interaction point $\bar {\boldsymbol {r}}_{i,l}$) from the set of cells that the $i^{th}$ incident beam passes through. The interaction point $\bar {\boldsymbol {r}}_{i,l}$ is taken to be located at the middle of the discretized segment in the $l^{th}$ cell. The inner sum in Eq. (6) computes numerically the spatial integral in Eq. (1) (integral over $\mathbf {r}$) using a Riemann sum. The two attenuation functions $f$ and $h$ are computed numerically using Riemann sums to approximate the integrals in Eqs. (2) and (3), giving

\begin{align*} f(\bar {\boldsymbol {r}}_{i,l},\boldsymbol {r}_{S,i},E_{S_{k}})=&\exp \left ({-\boldsymbol {a}_{i,l}^{T} \boldsymbol \mu (E_{S_{k}})}\right)\tag{7}\\ h(\boldsymbol {r}_{D',j},\bar {\boldsymbol {r}}_{i,l},E'_{i,j,l,k})=&\Omega _{D'}\exp \left ({-\boldsymbol {b}_{i,j,l}^{T} \boldsymbol \mu (E'_{i,j,l,k})}\right)\tag{8}\end{align*}

where $\boldsymbol {a}_{i,l}^{T}$ is a row vector with elements ordered lexicographically from a matrix the size of the discretized grid. This vector stores zeros for elements corresponding to cells that do not intersect with the incident beam connecting $\boldsymbol {r}_{S,i}$ to $\boldsymbol {r}_{i,l}$, and otherwise stores the length of the intersected beam segment across each cell. In the same manner, the vector $\boldsymbol {b}_{i,j,l}^{T}$ stores lengths along the scattered ray path from the interaction point $\boldsymbol {r}_{i,l}$ to the secondary detector $\boldsymbol {r}_{D',j}$. Moreover, $\boldsymbol \mu (E_{S_{k}})$ and $\boldsymbol \mu (E'_{i,j,l,k})$ are vectors with lexicographically-ordered elements that store the attenuation function (Eq. (4)) evaluated over the discretized cells, assuming energies of $E_{S_{k}}$ and $E'_{i,j,l,k}$, respectively. The subscripts on $E'_{i,j,l,k}$ indicate that the energy shift is a function of the scattering geometry.

Referring back to Eq. (6), $\theta _{i,j,l}$ in the scattering function $S(\bar {\boldsymbol {r}}_{i,l,},\theta _{i,j,l},E_{S_{k}}) $ is the scattering angle that lies between $i^{th}$ incident beam and scattering beam connecting $l^{th}$ interaction point to $j^{th}$ secondary detector. On the other hand, the weighting function $\omega (i,j,l,k,m) $ is used to approximate the Dirac delta function $\delta _{E_{D}}(E') $ in Eq. (1) as:

\begin{equation*} \omega (i,j,l,k,m) = \begin{cases} 1, & \text {such}~\text {that}~E'_{i,j,l,k} \in \\ & \left [{E_{m}-\frac {\Delta E}{2},E_{m}+\frac {\Delta E}{2} }\right], \\ 0, & \text {else}. \end{cases} \tag{9}\end{equation*}

The function $\delta _{i,l}$ in Eq. (6) approximates $\delta _{\boldsymbol {r}_{D},\boldsymbol {r}_{S}}(\boldsymbol {r}) \mathrm {d}\boldsymbol {r}$ in Eq. (1) by storing the segment length of $i^{th}$ incident beam intersecting with $l^{th}$ interaction cell. $\rho (\bar {\boldsymbol {r}}_{i,l,})$ is the discretized value of the density for the cell located at $\bar {\boldsymbol {r}}_{i,l,}$.

Eq. (6) computes the photon counts before interaction with the detector. A discrete form of the detector sensitivity function can be found by computing a detector response matrix $\mathbf {S}$ (described in [6] for the Multix detectors used in our experiment). If the detector has $Q$ output energy channels, then $\mathbf {S}$ is of size $Q \times N_{D}$ and its entries are $\mathbf {S}_{q,m} = s_{q}(E_{m})$. The signal $\boldsymbol {g}_{D'}(i,j,m)$ after the detector interaction occurs is computed as:

\begin{equation*} \boldsymbol {g}_{D'}(i,j,q)= \sum _{m} \boldsymbol {S}_{q,m} \boldsymbol {g}_{S}(i,j,m)\tag{10}\end{equation*}

### C. Forward Model for Attenuation Data

The forward model for attenuation data is considerably simpler than the Compton model, and builds closely on the notation above. In subsequent discussion, we will also refer to attenuation data as transmission or ‘Tx’ data. For a raypath between a source-detector pair $(\boldsymbol {r}_{S}, \boldsymbol {r}_{D})$, the continuous model for the number of counts collected on detector energy channel $q$ is:

\begin{equation*} g_{A}(\boldsymbol {r}_{D},\boldsymbol {r}_{S},E_{q}) = \int s_{q}(E_{S}) I(E_{S}) f(\boldsymbol {r}_{D},\boldsymbol {r}_{S},E_{S}) dE_{S}\tag{11}\end{equation*}

where $f(\boldsymbol {r}_{D},\boldsymbol {r}_{S},E_{S})$ has the same form as defined in Eq. (2), but has a raypath that extends from $\boldsymbol {r}_{S}$ to $\boldsymbol {r}_{D}$. All other terms are as defined above.

Similarly, the discretized version of the forward model follows closely from Eq. (7) and Eq. (10). Eq. (7) becomes for the attenuation case:

\begin{equation*} f(\bar {\boldsymbol {r}}_{i},\boldsymbol {r}_{S},E_{S_{k}})=\exp \left ({-\boldsymbol {a}_{i}^{T} \boldsymbol \mu (E_{S_{k}})}\right)\tag{12}\end{equation*}

where $i$ denotes the raypath. $\boldsymbol {a}_{i}^{T}$ and $\boldsymbol \mu (E_{S_{k}})$ are row vectors defined as in Eq. (7). Eq. (12) differs from Eq. (7) only in that there is no need to track the intersection point. The multiplication of $\boldsymbol {a}_{i}^{T}$ and $\boldsymbol \mu $ implements the integral along the raypath as a Riemann sum.

When processing dual- or multi-energy X-ray attenuation data, a common approach is *sinogram decomposition*, in which the measured projections are decomposed into the two energy-independent terms shown in Eq. (4) (see [9] and references therein). In this representation, the spatial integral in Eq. (2) is rewritten as:

\begin{equation*} \boldsymbol {a}_{i}^{T} \boldsymbol \mu (E_{S_{k}}) = c_{\tilde {\rho },i} f_{KN} (E_{S_{k}}) + c_{p,i} f_{p}(E_{S_{k}})\tag{13}\end{equation*}

where $c_{p,i} = \boldsymbol {a}_{i}^{T} \boldsymbol {p}$ is the integral of photoelectric absorption along beam $i$, where $\boldsymbol {p}$ is a column vector of size $N_{Cells} \times 1$ that contains the lexicographically unwrapped values of the photoelectric absorption in each cell. Similarly, $c_{\tilde {\rho },i} = \boldsymbol {a}_{i}^{T} \tilde {\boldsymbol \rho }$ integrates a scaled version of the density along the raypath, with the scaled density defined as $\tilde {\rho }(\boldsymbol {r})=\frac {\mathrm N_{A}}{2}\rho (\boldsymbol {r})$, where we have taken advantage of the fact that $\frac {Z(\boldsymbol {r})}{A(\boldsymbol {r})} \approx 1/2 $ for most elements [22].

This formulation of the forward model is convenient as data can be collected at various energies and used to estimate $c_{\tilde {\rho },i}$ and $c_{p,i}$, solving a separate nonlinear inverse problem for every raypath $i$. Details of the sinogram processing are described in Appendix B and as well as in [9]. The result of this processing are estimates of the projected density and photoelectric coefficients along each raypath. These projections are related to the underlying data through a system matrix $\boldsymbol A$ of size (# raypaths x $N_{Cells}$) whose rows are the vectors $\boldsymbol {a}_{i}^{T}$ already defined:

\begin{align*} \boldsymbol c_{\tilde {\rho }}=&(\mathrm N_{A} / 2) \mathbf {A} \boldsymbol \rho \\ \boldsymbol c_{p}=&\boldsymbol A \boldsymbol p\tag{14}\end{align*}

Thus, once sinogram decomposition is performed, recovery of the density and photoelectric images is a straightforward linear inverse problem. In the overall inversion method described below, we seek to solve this linear inverse problem as part of a joint inversion that combines attenuation and scattered data.

### D. Reconstruction Algorithm

Eq. (10) above gives an expression for the scattered data for source $i$, receiver $j$ and energy $q$. Collecting the results for all $i,j$ and $q$ into a vector $\boldsymbol {g}_{D'}$, the forward model for the Compton scattering signal described in Sections II-A and II-B can be represented as:

\begin{equation*} \boldsymbol {f}(\boldsymbol \rho,\boldsymbol p)=\boldsymbol {g}_{D'}\tag{15}\end{equation*}

where $\boldsymbol {f}(\boldsymbol \rho,\boldsymbol p)$ is the nonlinear model of the Compton scattering signal as a function of both the density $\boldsymbol \rho $ and the photoelectric absorption coefficients $\boldsymbol p $. To avoid confusion with the signal $\boldsymbol {g}_{D'}$ computed using the model above, the actual experimental data recorded from the instruments will be referred to as $\boldsymbol d$. Similarly, in place of $\boldsymbol c_{\tilde {\rho }}$ and $\boldsymbol c_{p}$ from Eq. (14), $\boldsymbol s_{\tilde {\rho }}$ and $\boldsymbol s_{p}$ will refer to the sinogram decomposition of the density and the photoelectric coefficient, extracted from the actual transmission data.

For density reconstruction, the optimization function can be written as:

\begin{align*}{rCl} \hat {\boldsymbol \rho }=&\mathop {\arg \min }\limits _{\boldsymbol \rho } \Biggl \{0.5\gamma ^{sc} \left \|{ \boldsymbol {f}(\boldsymbol \rho,\boldsymbol p_{(0)})-\boldsymbol d}\right \| _{2}^{2} \\&+\,0.5\gamma ^{tx} \left \|{ (\mathrm N_{A} / 2) \mathbf {A} \boldsymbol \rho -\boldsymbol s_{\tilde {\rho }} }\right \| _{2}^{2} \\&+\,\lambda ^{TV} \left |{\nabla \boldsymbol \rho }\right |_{1} \Biggr \}\tag{16}\end{align*}

where $\gamma ^{sc} \le 1$ and $\gamma ^{tx} \le 1$ are weights used to control the relative importance of data misfit terms for scattering data (first term) and attenuation data (second term). The enforcement of the TV regularization is controlled through the $\lambda ^{TV}$ parameter. Higher $\lambda ^{TV}$ will encourage reconstructed profiles that are piecewise constant with clearly defined edges, a widely used regularization strategy in attenuation-only computed tomography [28]–[30]. Equation. (16) is subjected to an additional constraint ${\boldsymbol \rho }\ge 0$, i.e., negative densities are not allowed.

The formulation above lets us study the relative impact of scattering and attenuation data on our solutions. In this work, we will consider four scenarios to study the optimization problem in Eq. (16), namely i) inversion using Compton scatter data only ( $\gamma ^{sc}= 1, \gamma ^{tx}= 0$), ii) inversion using attenuation data only ( $\gamma ^{sc}= 0, \gamma ^{tx}= 1$), iii) inversion equally weighting both data types ( $\gamma ^{sc}= \gamma ^{tx}= 1$), and iv) and inversion that weights scattering data more heavily than attenuation data ( $\gamma ^{sc}= 1, \gamma ^{tx} < 1$).

**FIGURE 3.**
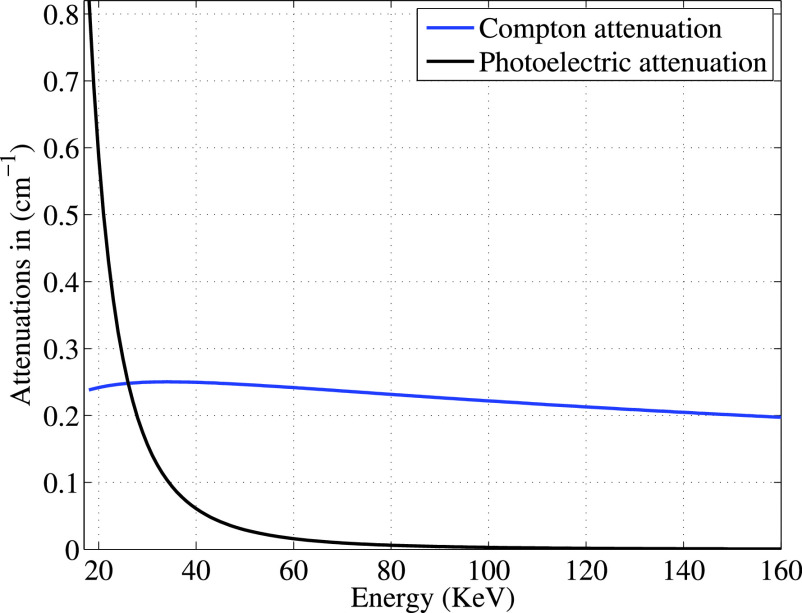
Photoelectric and compton attenuations of delrin.

The optimization function in Eq. (16) is written in term of $\boldsymbol \rho $ only, so an estimate of the photoelectric coefficients is required. Although the Compton scattering signal as described in Eq. (6) depends nonlinearly on both $\boldsymbol \rho $ and $\boldsymbol p$, we can exploit the fact that the higher energy attenuation is dominated by $\boldsymbol \rho $. Fig. 3 plots the two portions of the attenuation function (see Eq.(4)) due to the Compton scattering term ( $N_{A} (Z(\boldsymbol {r})/A(\boldsymbol {r}))f_{KN}(E)\rho (\boldsymbol {r})$ and due to the photoelectric absorption term ( $f_{p}(E)p(\boldsymbol {r})$) for Delrin, a plastic material used in our experimental work. The figure shows that the photoelectric absorption effect becomes increasingly negligible above 40KeV; similar results are seen for other materials as photoelectric absorption falls as the inverse cube of energy. Hence, in our density reconstructions, we neglect the photoelectric effect (set $\boldsymbol p=0$ in Eq. (16)) and measure data misfit for scattered data for higher energy photons only. While multiple higher-energy bins could be used, in the results below we sum all higher-energy scattered photons into a single energy channel. Note that this approach requires the use of X-ray detectors with the ability to resolve detected photon energy.

For the reconstruction process, the following algorithm shows how the optimization in Eq. (16) can be carried out using a steepest descent approach [25], [31], [32], [32]–[37] known as embedded nonlinear Landweber-Kaczmarz algorithm [25], [36], [37]

\begin{equation*}\begin{array}{l} {{ Algorithm\;I}} \\ {Step\;1.0:{{ \; Initialize\;}} \boldsymbol \rho _{0}= 0,\boldsymbol p_{0} = 0} \\ {Step\;2.0:\; Set \; parameters\; \gamma ^{sc},\gamma ^{tx},\lambda ^{TV},\beta } \\ {Step\;3.0:\; for\; i=0,1,\ldots,N^{iter} } \\ {Step\;3.1:\; \; \; \; \; \; \; \; \boldsymbol \rho _{i+1} =\boldsymbol \rho _{i}} \\ {\; \; \; \; \; \; \; \; \; \; \; \; \; \; \; \; \; \; \; \; \; \; \; \; \; \; \,\,-\gamma ^{sc} \omega _{i}^{sc} \partial _{\boldsymbol \rho _{i} } \boldsymbol {f}\left ({\boldsymbol \rho _{i},\boldsymbol p_{0} }\right)^{T} \left ({\boldsymbol {f}\left ({\boldsymbol \rho _{i},\boldsymbol p_{0} }\right)-\boldsymbol d}\right)} \\ {\; \; \; \; \; \; \; \; \; \; \; \; \; \; \; \; \; \; \; \; \; \; \; \; \; \;\,\, -\gamma ^{tx} \omega ^{tx} (N_{A} / 2) \mathbf {A} ^{T} \left ({(N_{A} / 2) \mathbf {A} \boldsymbol \rho _{i} -\boldsymbol s_{\tilde {\rho }} }\right)} \\ {\; \; \; \; \; \; \; \; \; \; \; \; \; \; \; \; \; \; \; \; \; \; \; \; \; \;\,\,-\lambda ^{TV} \nabla.\left ({\frac {\nabla \boldsymbol \rho _{i}}{\sqrt {\left |{\nabla \boldsymbol \rho _{i} }\right |^{2} +\beta } } }\right)} \\ {Step\;3.2:\; \; \; \; \; \; \; \; \boldsymbol \rho _{i+1} =max(0,\boldsymbol \rho _{i})} \\ {{ Step\;3.3:\; end\; loop}} \end{array}\end{equation*}

While most of the notation above has already been introduced, we note that $\beta $ is a small number used to remove the derivative singularity in the TV regularization term, $N^{iter}$ is the number of gradient descent iterations, and $\partial _{\boldsymbol \rho _{i} } \boldsymbol {f}\left ({\boldsymbol \rho _{i},\boldsymbol p_{0} }\right)$ is the adjoint of the density differential, a matrix that is described in detail in Appendix A which relates each measurement to the change in density at every point. In the algorithm, Step 1.0 initializes the density profile $\boldsymbol \rho _{0}$ to zero and sets the photoelectric profile $\boldsymbol p_{0}$; note that the density profile is updated in Step 3, while photoelectric values remain at zero. In Step 2.0 the user sets the weighting parameters $\gamma ^{sc} $ and $\gamma ^{tx} $ of the Compton and transmission data misfits, respectively, and sets the TV regularization parameters. Step 3.0 is the iteration loop of the nonlinear Landweber algorithm with $N^{iter}$ iteration steps. Step 3.1 shows one step of the Landweber iteration over the density $\boldsymbol \rho $, where $\gamma ^{sc} \omega _{i}^{sc}$ and $\gamma ^{tx} \omega _{i}^{tx}$ defines the step sizes per iteration for scattering and transmission updates, respectively. A sufficient condition for convergence of nonlinear Landweber-Kaczmarz states that $\gamma ^{sc} \omega _{i}^{sc} \le 1/(\sigma ^{sc})^{2}$ and $\gamma ^{tx} \omega _{i}^{tx} \le 1/(\sigma ^{tx})^{2}$ where $\sigma _{i}^{sc}$ and $\sigma ^{tx}$ are the maximum singular values of $\partial _{\boldsymbol \rho _{i} } \boldsymbol {f}\left ({\boldsymbol \rho _{i},\boldsymbol p_{0} }\right)$ and $(\mathrm N_{A} / 2) \mathbf {A}$ respectively, [36], [37]. Since $\gamma ^{tx} \le 1$ and $\gamma ^{sc} \le 1$, to achieve maximum possible step sizes under the convergence condition, $\omega _{i}^{tx}= 1/(\sigma ^{tx})^{2}$ and $\omega _{i}^{sc}= 1/(\sigma ^{sc})^{2}$ are assumed. The upper script $(^{T})$ is used to indicate the transpose operator. Finally, Step 3.2 enforces the nonnegativity constraint on density [38].

### E. Computational Considerations and Implementation

The maximum step size for the Landweber algorithm is set by $\omega _{i}^{sc}$ and $\omega ^{tx}$, which in turn depend on the maximum singular values $\sigma _{i}^{sc}$ and $\sigma ^{tx}$ for the sensitivity operators related to scattered data and attenuation data [33], [35], [37]. These maximum singular values are computed using power iteration [39]. While $\sigma ^{tx}$ is a fixed quantity as the transmission model is a linear model, $\sigma _{i}^{sc}$ changes each time the nonlinear Compton scattering model is updated. The computation of $\sigma _{i}^{sc}$ is expensive as it requires evaluating the differential and the adjoint operators at least four times each. However, it is not necessary to re-evaluate $\sigma _{i}^{sc}$ on each iteration, due to the fact that the nonlinear Landweber will have its largest changes in the first iteration steps, with changes in $\sigma _{i}^{sc}$ becoming smaller as iteration proceeds. Hence for the results below, $\sigma _{i}^{sc}$ will be only evaluated for selected iteration steps. For all the examples, $\sigma _{i}^{sc}$ is computed at the first three iteration steps, then at every tenth step up to iteration 50, then again at iterations 75, 100 and 150.

The computation at each iteration is dominated by calculating the forward model Eq. (6), the differential $\partial _{\boldsymbol {\rho }} \boldsymbol {f}(\boldsymbol {\rho },\boldsymbol {p})$ Eq. (26), and the adjoint $\partial _{\boldsymbol {\rho }} \boldsymbol {f}(\boldsymbol {\rho },\boldsymbol {p})^{T}$ Eq. (27) operators. Each of these terms is a matrix indexed by the incident source beam, $i$, and secondary detector, $j$. Computing each element of these matrices involves integrations over spatial cells and photon energies, rapidly increasing the dimensions of the problem and leading to a computational challenge. Fortunately, these calculations (described in Appendix A) lend well to parallelization across beam/detector pairs.

Initially the reconstruction algorithm was fully implemented in Matlab 2016a and run on a High Performance Computing cluster at Tufts University. Calculation of the elements of the forward model, differential, and adjoint matrices were each parallelized by splitting the elements evenly across nodes using Matlab’s Parallel Processing Toolbox. Reconstructions for the scan scenario and test images discussed in Section III.C typically required 3–5 days on up to 20 compute nodes (the number and type of cluster nodes available varied between runs).

To allow faster processing times, the forward model, differential, and adjoint calculations were translated to C and parallelized for a single node using OpenMP. The Intel *icc* compiler was used with O3-level optimization and AVX2 flags. Further acceleration was achieved by improving the load balancing across processors. Note from Figure 2 that different incident beams generally traverse over a different number of voxels in the investigation domain. This means that computing the integrations over voxels for different source/detector pairs require different amounts of computation. Rather than splitting the computation by assigning each processor a fixed number of operator elements, prior knowledge of the geometry allows the workload to be split more evenly thus achieving a better load balance. Running the overall reconstruction within Matlab, but escaping to the compiled routines for the three major operators, resulted in equivalent reconstructions that completed on the order of 9 minutes on a single dedicated server with two 20-core Xeon E5-2698v4 processors.

## III. Experimental Results

In this section, experimental results are illustrated to verify the forward model and to study reconstruction results. We describe the exeperimental testbed, discuss calibration and validation of the forward model, then show reconstruction results for several scenarios.

### A. Experimental Testbed

**FIGURE 4.**
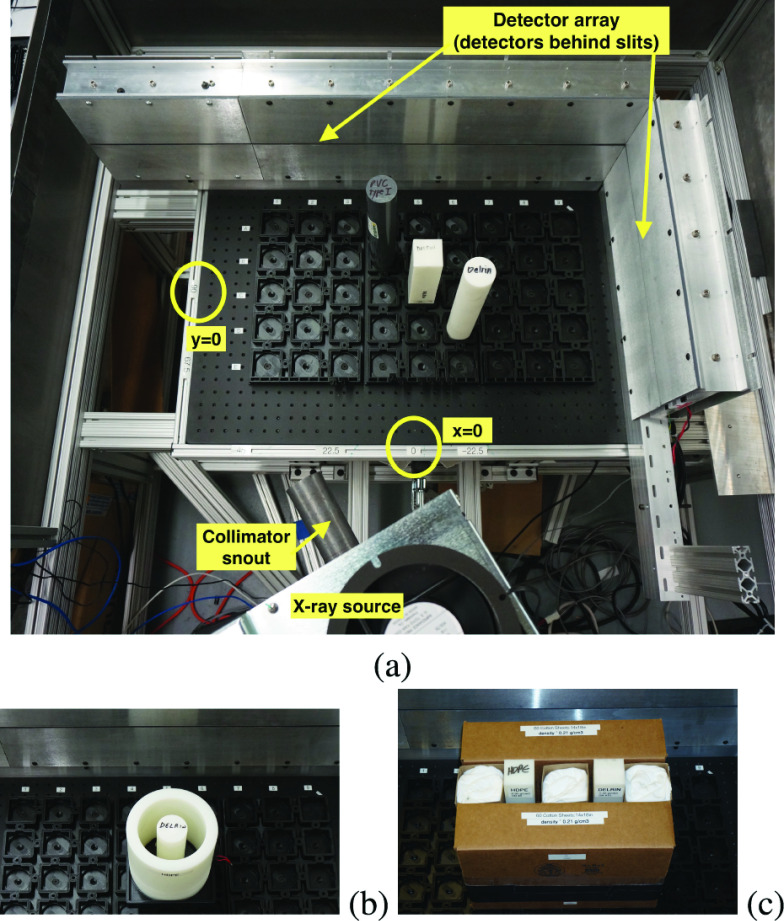
Experimental setup for measurements; a) top view showing the X-ray source, source collimator, and L-shaped array, three-target scenario (reconstructions in Fig. 8); markers show the origin of $(x,y)$ coordinate system. A slit opening built into the detectors ensures that only in-plane scatter is measured; b) zoom on coaxial target (reconstructions in Fig. 9); c) zoom on box target (reconstructions in Fig. 11).

A multi-energy X-ray testbed system was constructed to experimentally measure both transmission (Tx) and Compton scattering data in a controlled environment using energy-discriminating detectors. This testbed, shown in Fig. 4, consisted of an L-shaped detector array mounted on an optical table (L-shaped detector arrays are the norm for conventional transmission X-ray baggage scanners, as they make efficient use of space for the rectangular tunnels used in these scanners). A roughly 60 cm $\times40$ cm section of the optical table was outfitted with regular 2” $\times2$” indentations that allow the repeatable positioning of material samples and other image targets. Both 2” square and 2” diameter circular test objects could be mounted directly to the testbed. Mechanical adaptors were built to allow for repeatable positioning of larger objects.

*Detectors*: The detector array consisted of ME-100 photon-counting detectors (Multix, Inc., Moirans, France), arranged in 14 modules each containing 128 detectors. Five modules (640 detectors) were arranged on one side of the L with 9 modules (1152 detectors) on the other, giving 1792 detectors in total. Each detector pixel was approximately 0.08 cm in width and height. A lead collimator is built into the detector package to suppress scatter from objects outside of the plane formed by the X-ray source and detector array. An additional radiation shield, shown in Fig. 4, further reduces scatter from outside the detector plane. During data collection, detectors were operated at the finest energy resolution, outputting 128 evenly spaced energy channels from 20–160 keV. This represents an over-sampling, as the actual energy resolution of the ME100 is closer to 5–7 keV, depending on energy. A detailed model of the ME100 energy resolution, based on [6], was used in reconstruction.

**FIGURE 5.**
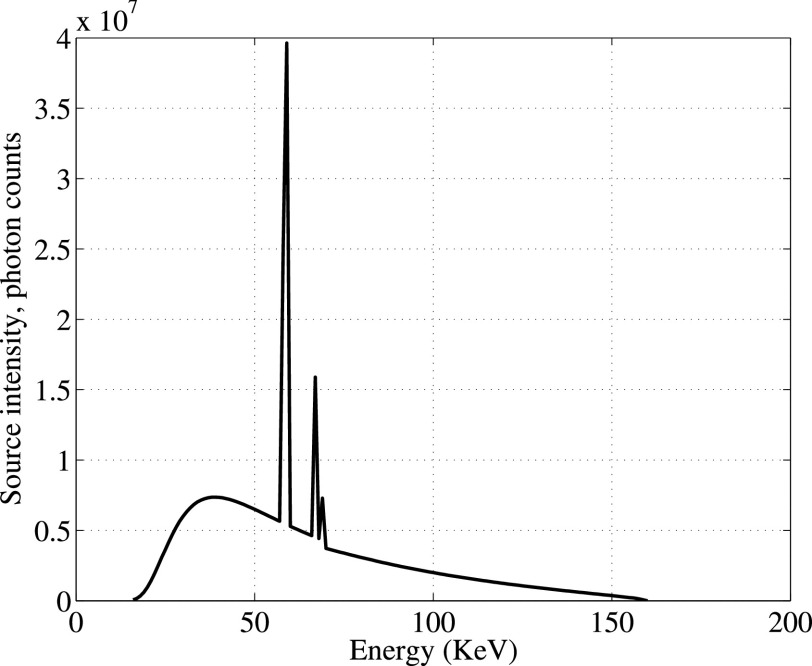
Source spectrum intensity.

*Source*: The objects were interrogated using a single X-ray source (bremsstrahlung spectrum with peak energy 160 keV and average energy 62 keV, giving the spectrum in Fig. 5). A source collimator was used to produce a narrow pencil beam, as discussed in detail below. The source intensity (shown in Fig. 5) was computed pre-collimation, leading to a need to calibrate the emitted source flux, with calibration described in the next section. This source was mounted on a pivot at a radius of 76.2 cm from the origin of the coordinate system, which was taken to be in the middle of the interrogation region (see Fig. 4(b)). The testbed could be precisely configured to interrogate the scene from 6 different angles spaced apart by 22.5°, ranging from −22.5° to 90°, with the 0° source perpendicular to the longer detector array (source position ( $x=0,y=-76.2$ cm) and the 90° source perpendicular to the shorter detector array (source position ( $x=-76.2$ cm, $y=0$). For each source position, the source was rotated so beams were swept across the interrogation domain in 0.4° steps. Data from the various source positions were collected to mimic a multi-source system in which sources scan the region sequentially.

**FIGURE 6.**
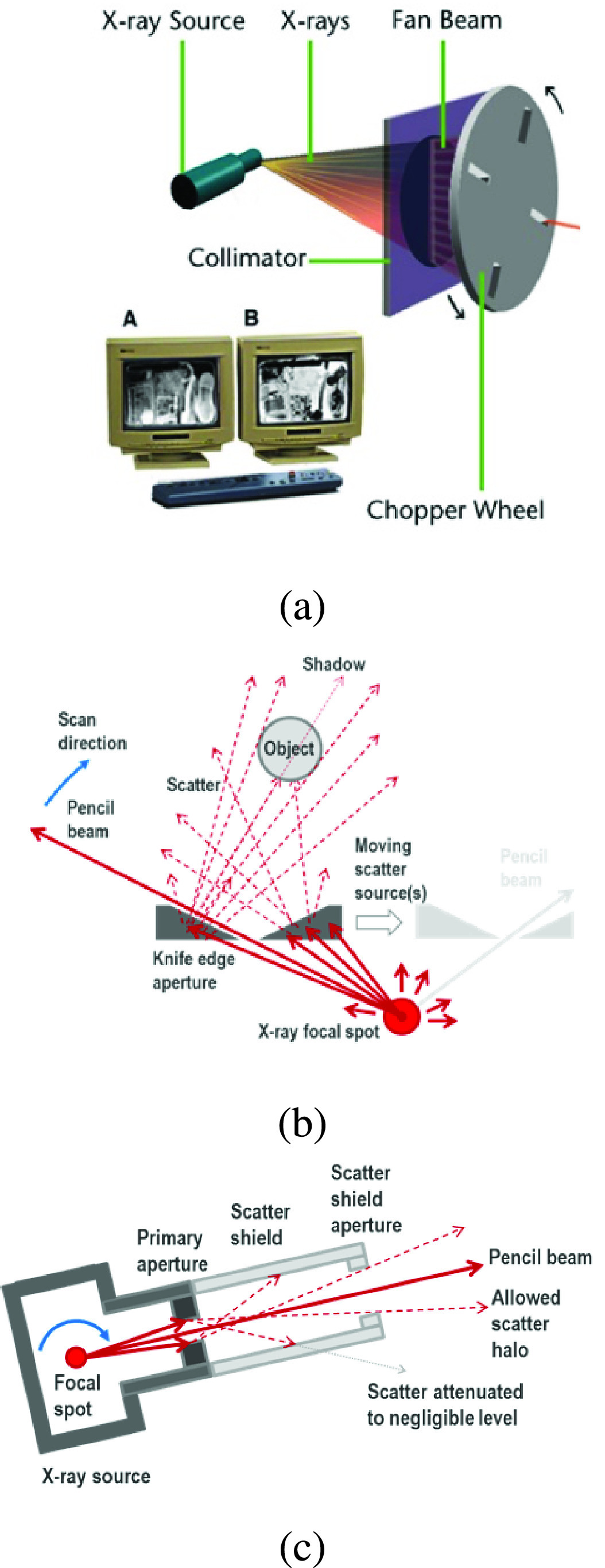
Source collimation alternatives tested during experimentation. a) shows a conventional chopper system for creating a pencil beam; b) shows artifacts generated by scattering off the conventional chopper aperture; c) shows the rotating source and snout concept, with a shield to limit scatter from the primary aperture.

Initial experimental results showed that scatter from the pencil beam forming collimator itself was discovered to be a major limiting factor, which resulted in several iterations of hardware development. The original design employed a pencil beam forming system that is standard in commercial X-ray backscatter systems [40], shown in Fig. 6(a). The cone beam from the X-ray source is chopped into a pencil beam by a combination of a rotating disk with radial slot apertures and stationary pre-collimator slot in the plane of the detectors. As the disk rotates, the combination of disk aperture and pre-collimator form a rhombus shaped opening which sweeps across the field of view. Unfortunately, a rotating disk chopper requires a knife edge aperture, as shown in Figure 6(b),which created substantial scatter and fluorescence, both of which added noise to the overall data. Furthermore, because the point of scatter moves as the disk rotates, a complex set of systematic errors were embedded in the data, which included shifting transmission images of any objects in the tunnel.

To avoid these artifacts, the final design employed a snout which was fixed to the X-ray source, and the entire assembly of source and snout rotated to sweep the pencil beam across the tunnel, as shown in Fig. 6(c). The direct connection of snout to source results in containment of leakage radiation and a uniform pencil beam cross section. More importantly, this geometry controlled the scatter from the apertures themselves. Monte Carlo simulations predicted that simply replacing the knife edge aperture with a simple tunnel should reduce scatter and fluorescence by 10 $\times$–20 $\times$. The inclusion of a scatter shield further limited unwanted scatter in all directions except for a small region near the primary pencil beam. Collimation by the ‘primary aperture’ forms the pencil beam. The primary aperture itself is positioned only as far from the focal spot as deemed necessary to provide adequate spatial resolution (i.e. to adequately limit of the divergence angle of the pencil beam.) Most of the length of the snout serves as a scatter shield to contain scatter from the primary aperture. The exit opening of the snout defines a narrow angle of allowed scatter which is effectively a halo around the pencil beam. Direct beam from the halo illuminates a few dozen pixels on either side those in the path of the good pencil beam. Compton interactions from the narrow halo are orders of magnitude fewer than from the unwanted scatter in the original system, which was critical to obtaining a good match between forward model and experimental data. All data results shown in this paper were generated using this snout design.

### B. Calibration and Forward Model Verification

The X-ray source used to generate the incident beam has intensity $\mathrm I_{S}(E_{S})$ shown in Fig. 5. Due to the source collimation, a calibration parameter $\alpha _{c}$ is required to determine the effective photon counts on the incident beam $\mathrm I(E_{S})$ in Eq. (1), such that $\mathrm I(E_{S})= \alpha _{c} \mathrm I_{S}(E_{S})$. The parameter $\alpha _{c}$ was determined by matching the forward model to data collected using a reference object (a 2” cylinder of known density) with the beam pointed at the center of the object. After collecting the data, high noise detectors were dropped and the predicted data for the remaining detectors was computed using the model described in Section II-A with $\mathrm I(E_{S})=\mathrm I_{S}(E_{S})$. The computed curve was then multiplied by the unknown $\alpha _{c} $ and matched with the data using least squares minimization.

**FIGURE 7.**
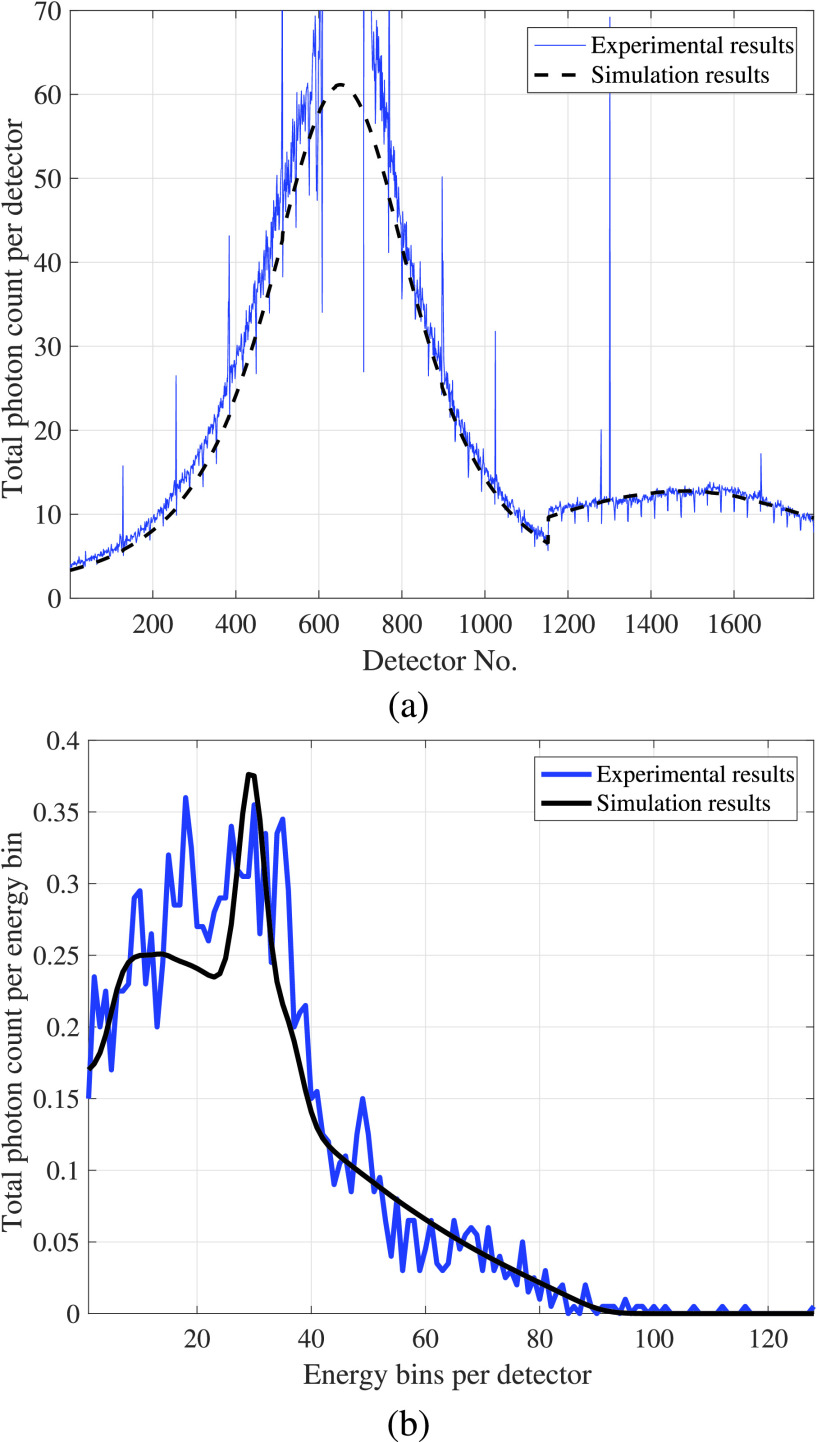
Forward model verification, showing (a) total photon counts per detector, and (b) photon counts per energy bin at detector 1550. Detectors 1–1152 are for the upper side of the detector (see Fig. 4), and the jump in figure (a) at detector 1153 marks the transition between sides of the L-shaped detector array.

Example output from the resulting calibrated model is shown in Fig. 7. A Delrin (CH_2_O) target of 2.54 cm radius was placed at the center of the experimental fixture. The X-ray source was located at 0° source (see description in Section III-A) with the beam directed toward the center of the object. Fig. 7(a) shows the sum of photon counts over all the energies at each detector for both experimental and simulation results. In the experimental results, the photons are collected over 0.1 msec time slots and are then averaged over 200 time slots for a total observation time of 20 sec. The jump at detector 1153 marks the transition from the longer to the shorter arms of the L-shaped detector array (shown in Fig. 4). Fig. 7(b) shows the number of photon counts per energy at a single detector (number 1550), for both simulation and experiment. The effect of statistical noise can be clearly seen in Fig. 7(b), as the number of photons per energy bin on one detector is relatively low. However, in general the forward model in Fig. 7 shows a close match to the experimental results. Note that spectrum shape in Fig. 7(b) is smoothed compared to the source spectrum (Fig. 5) due to the imperfect energy resolution of the detectors [6].

While the overall match in Fig. 7(a) is good, isolated detectors are seen that have anomolously high or low counts relative to their neighbors. Detectors are arranged in blocks of 32, and investigation showed that a majority of anomolous detectors were located at the edges of each detector block, while other anamolous detectors could be identified by measuring dark-scan current (i.e., detector output with the X-ray source turned off). Anomalous detectors were identified and removed before further processing.

### C. Reconstruction Results

To test image reconstruction, a series of phantoms objects were measured on the testbed. These phantoms range from simple geometric objects to objects more typical of airport luggage. In this section, we present reconstuction results for three scenarios: a) an arrangement of three plastic rods; b) a coaxial plastic object with an inner Delrin cylinder surrounded by an outer HDPE cylinder; and c) a cardboard box packed with cotton T-shirts that concealed Delrin and HDPE square rods. As described above, detector collimation was used to ensure that only in-plane scattering was measured, so all reconstructions are two-dimensional.

Data were collected from all 6 source locations. Because we seek to understand the benefit of Compton scattering data for few-view systems, we processed data for configurations of 3, 4 and 6 sources. The three-source configuration included the (90°, 45°, −22.5°) sources, while the 4-source configuration used the (90°, 45°,0°, −22.5°) sources. Reconstructed images are plotted below only for selected source configurations, but image quality metrics for a wider set of configurations are found in Table 1.

**TABLE 1:** Quantitative Comparison of Methods. First Number is SSIM, Second Number is Normalized Error (Both Computed vs. Nominal Density). High SSIM and Low Errors are Desired; Best Result for Each Case is Bolded

	Compton	Tx	Compton+Tx
3 targets, 3 src	0.23 / 263.1	0.29 / 78.4	0.36 / 63.7
3 targets, 4 src	0.25 / 228.6	0.29 / 72.1	0.39 / 62.3
3 targets, 6 src	0.27 / 221.2	0.31 / 66.4	0.40 / 62.3
Coax, 3 src	0.18 / 305.5	0.26 / 108.0	0.35 / 96.2
Coax, 4 src	0.21 / 300.0	0.34 / 87.8	0.43 / 73.9
Coax, 6 src	0.21 / 290.1	0.38 / 75.5	0.45 / 69.9
Packed box, 6 src	0.36 / 214.9	0.52 / 44.4	0.49 / 41.2

A common set of parameters were used to reconstruct all scenarios shown. Reconstructions were generated on an evenly spaced grid (4 mm grid spacing in both dimensions) for 150 iterations of the algorithm.

Total variation parameters were set as $\beta =10^{-6}$ and $\lambda ^{TV} = 0.01$ for all examples. Selecting $\lambda ^{TV}$ is in general problem dependent. For optimization problems with “weighted” misfit terms that are function of their misfits, $\lambda ^{TV}$ should be updated to balance the optimization problem in accordance with the change in misfits weights [30]. The later makes selecting $\lambda ^{TV}$ a difficult task, so optimization schemes have been developed for such problems, e.g. the multiplicative constraint approach [38]. However, if the misfit terms are multiplied with fixed weights, as in Eq. (16), then selecting $\lambda ^{TV}$ becomes simpler and estimates can be found empirically [41], [42]. For *all* the examples illustrated in this work, a fixed value of $\lambda ^{TV} = 0.01$ is used.

Transmission sinogram decomposition was performed for all source-detector raypaths after elimination of bad detectors (as discussed in the previous section). Compton scattering measurements from good detector pixels were spatially averaged into 200 equivalent detectors. In effect, this simulates the use of larger-area scatter detectors (roughly $7x$ area increase), which helps to improve count statistics and to reduce computation time.

#### 1) Three Targets

**FIGURE 8.**
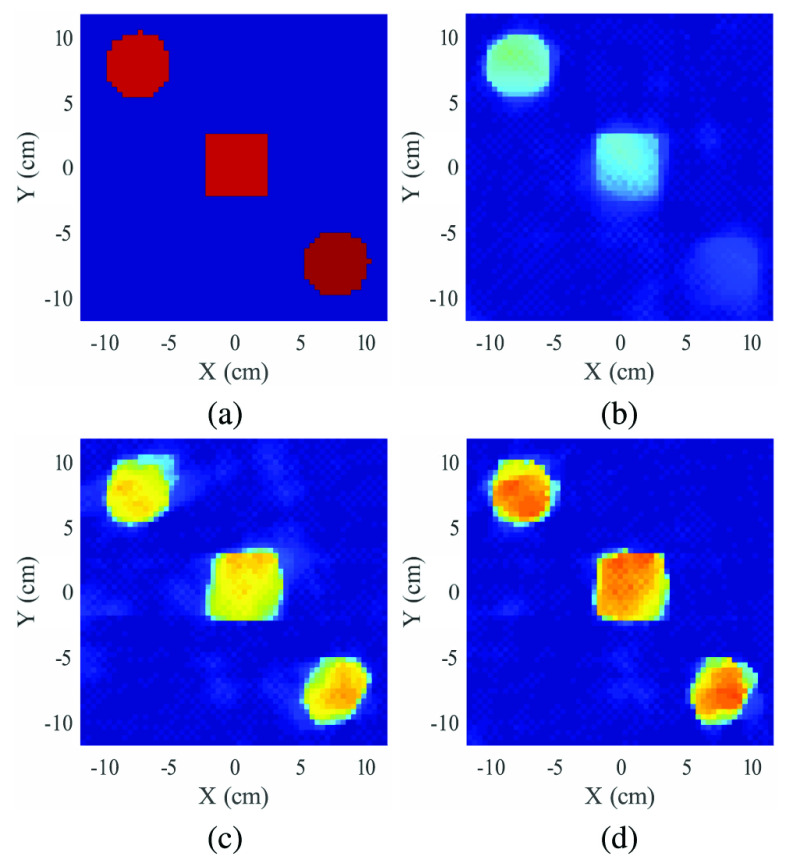
Reconstructed result of three targets example, showing (a) actual density profile, (b) reconstruction using Compton scattering data only, (c) reconstruction using Tx data only, (d) reconstruction using both Compton and Tx data. All density reconstructions are shown for a dynamic range of 0 - $1.5~g/cm^{3}$.

In this example, the investigation domain contains three targets: a 2” diameter PVC cylinder (C_2_H_3_Cl), a 2” square Delrin rod (CH_2_O), and a 2” diameter Delrin cylinder (CH_2_O) (see Fig. 4). The results of density reconstructions for a 4-source configuation are shown in Fig. 8. Fig. 8(a) shows the nominal (ground truth) density. To simplify the plotting, this and all subsequent density reconstructions are shown for a fixed range of $0-1.5\,\,g/cm^{3}$ (note that PVC and Delrin densities are quite similar).

The remaining plots show reconstructed density using Compton-scattered data only (Fig. 8(b)), Tx data only (Fig. 8(c)), and both Tx and scatter data (Fig. 8(d)). For results in Fig. 8(d), equal weighting is placed on both Tx and scatter data (i.e., $\lambda ^{tx} = \lambda ^{sc} = 1$). The use of Compton scatter data alone yields a reconstruction with reasonable geometric detail, but with greatly underestimated density values. As a note, this result is the solution of a nonlinear, non-convex problem without convergence guarantees. The Tx-only reconstruction in subfigure (b) shows improved density estimates, but density is still underestimated, and there is a noticeable increase in artifacts (see blue background region). The combination of Tx and Compton scatter data in (d) shows the most accurate (highest) reconstructed density values, and also shows a reduction in artifacts relative to Tx-only results. The geometric definition of the three shapes is also improved as compared to Tx-only results. This improvement is attributed to the additional sampling of the scene provided by broken-ray raypaths.

Because a reliable estimate of the actual density is available, quantitative measures of the reconstructed image can be computed, using the nominal density image (subfigure (a)) as the reference image. Overall estimation error is measured using normalized mean-squared error (expressed as a percent), as well as the widely-used Structural Similarity Image Metric (SSIM) [43]. SSIM is most typically used as an image quality metric that matches human perception, which has relevance for an X-ray imaging system where images would be reviewed by a human operator. Both metrics are shown in Table 1 for all phantoms and various numbers of sources. Note that the combination of Tx and scatter data gives the lowest error for all cases, and the best SSIM for all but one case.

#### 2) Coaxial Geometry

**FIGURE 9.**
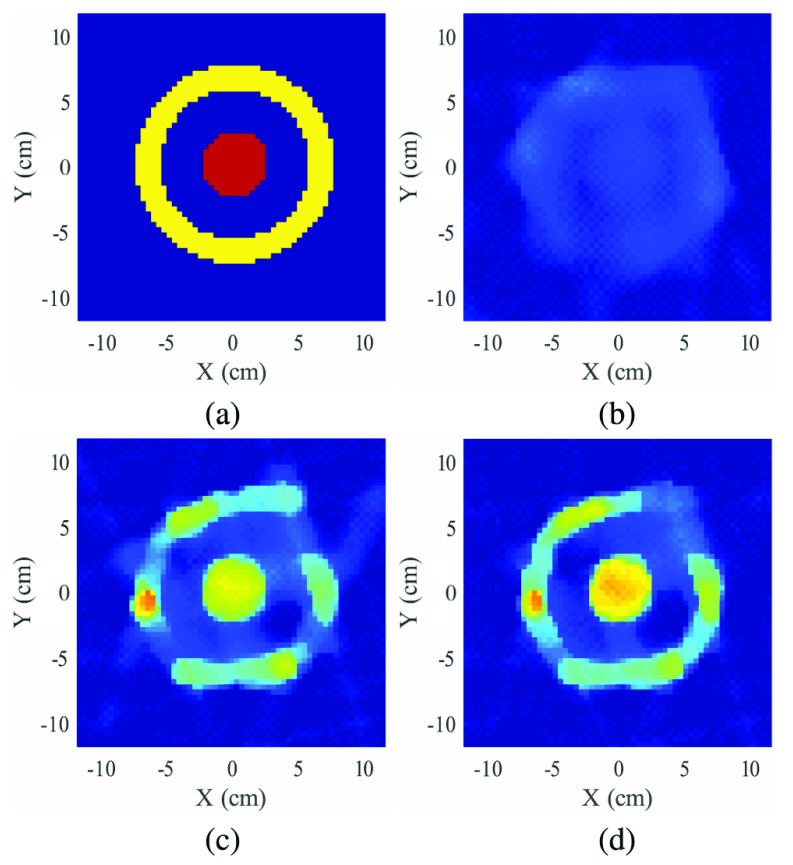
Reconstructed result of coaxial example, (a) actual density profile, (b) reconstruction using Compton scattering data only, (c) reconstruction using Tx data only, (d) reconstruction using both Compton and Tx data.

As a second example, we consider a coaxial geometry in which a Delrin cylinder is placed inside an outer HDPE shell. Ground truth and reconstruction results (again for the 4-source configuration) are shown in Fig. 9. The results are generally similar to the three-target phantom, with the combination of Tx and Compton-scattered data showing best results. While all reconstructions underestimate density, Fig. 9(d) shows more accurate values, particularly for the inner Delrin cylinder. In addition, the geometry of the outer cylinder is better defined, and artifacts outside the HDPE object are noticeably reduced.

An interesting feature of the Tx-only reconstruction in Fig. 9(c) is the noticeable variation in the reconstructed density of the outer ring. This is a result of the extremely limited number of angles used in reconstruction (the effect is reduced if all 6 sources are used). One potential gain from adding Compton scatter data is that the additional raypaths provide additional geometric information that can reduce artifacts in few-view reconstruction. A modest improvement is in fact visible, with the reconstructed HDPE ring being more homogenous in Fig. 9(d) than in Fig. 9(c). This leads to the question of whether Compton scatter data can be further exploited to reduce image artifacts.

**FIGURE 10.**
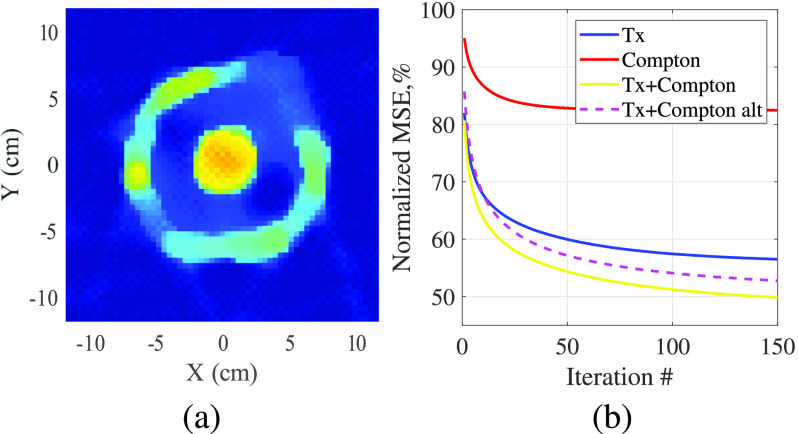
Alternate reconstruction, which emphasizes the role of Compton data during reconstruction by setting $\gamma ^{tx}=0.5$ and $\gamma ^{sc}=1$. (a) reconstructed density (b) percent norm error vs. nominal density for all methods. While the alternate reconstruction does not improve SSIM or overall error, it slightly improves homogeneity in the outer ring of the object.

As noted in Section II-D, convergence is guaranteed if the gradient descent steps based on Tx and scatter data are limited by choosing step size multipliers $\lambda ^{tx} \le 1$ and $\lambda ^{sc} \le 1$. In the results above, the largest possible steps were taken ( $\lambda ^{tx} = \lambda ^{sc}=1$) to accelerate convergence. However, given that the limited-view artifacts are primarily associated with transmission data, it is interesting to explore whether de-emphasizing Tx data can further reduce artifacts. Fig. 10(a) shows the result obtained by taking smaller steps based on transmission data, setting $\lambda ^{tx} = 0.5$ and $\lambda ^{sc}=1$. Careful visual inspection suggests some improvement in the image, particularly in the homogeneity of the outer ring. To quantify this, the ground truth image in Fig. 9(a) was used to identify all pixels corresponding to the outer ring. Variability of reconstructed density for these pixels was computed using percentile measures, with results shown in Table 2. There appears to be a modest but measurable improvement in homogeneity, with the most homogeneous reconstruction given by the result plotted in Fig. 10(a).

**TABLE 2:** Measures of Homogeneity in Coax Reconstruction, Focusing on Outer (HDPE) Ring. Variability is Measured as Distance Between the Percentile Values Shown, With the Lowest Variablity Shown in Bold

Reconstruction method	Inter-quartile range ( $75^{th}-25^{th}$ percentile)	$90^{th}-10^{th}$ percentile
Tx-only	0.320	0.550
Tx + scatter, equal weight	0.299	0.510
Tx + scatter, deweighted Tx	0.293	0.487

Fig. 10(b) compares the overall image error for various solutions as a function of iteration number, showing that varying the ratio $\lambda ^{tx} / \lambda ^{sc}$ changes the shape of the error curve *vs.* ground truth values. While in this instance the lowest overall error is given by equal weighting, determining the optimum weighting for transmission vs. scattered data in the inversion is a potentially interesting avenue for future work.

#### 3) Packed Box With Cotton and Targets

**FIGURE 11.**
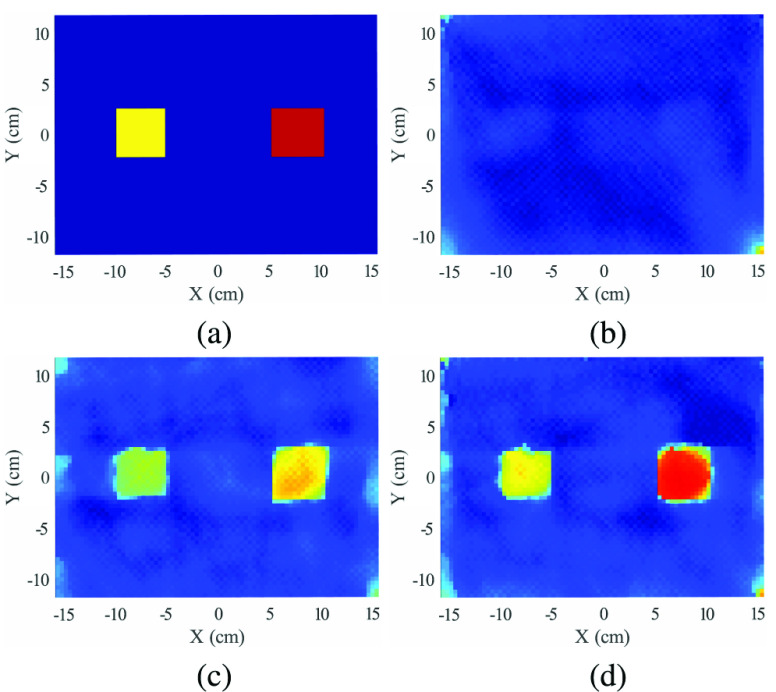
Reconstructed result of clutter example, (a) actual density profile, (b) reconstruction using Compton scattering data only, (c) reconstruction using Tx data only, (d) reconstruction using both Compton and Tx data.

As a final example, Fig. 11 shows a more ‘real-world’ scenario with more inhomogeneity in which polyethylene and Delrin objects are surrounded by several inches of cotton fabric (T-shirt material) and cellulose (the cardboard boxes used to contain the fabric.) This configuration approximates a cross section of a typical carry-on sized suitcase, packed with clothing and two masses of contraband organic material. The interior of the box is imaged, so the background density is that of cotton (taken to be $0.23~g/cm^{3}$). In this scenario, the Compton scatter-only result shows very weak contrast at the location of the two objects. The Tx-only result does recover the objects, but underestimates their density. The combination of Tx and scatter data yields the most accurate density estimates for the hidden objects, as shown by the normalized error listed in Table 1.

## IV. Discussion and Future Work

The work above has described physics-based models for both Compton scattering and transmission data, and used these models to reconstruct density of objects imaged by very few-view tomography systems. The experimental results showed that reconstructions that combined transmission (attenuation) data with Compton scatter measurements yield more accurate density estimates, and have reduced image artifacts, compared to transmission-only reconstructions. This suggests that current systems, which typically measure only transmission data and seek to eliminate scattered photons through collimation, are discarding potentially useful information that could improve image quality. A further result is that a first-order scattering model (neglecting potential multiple scattering) was sufficient for image recovery in our experiments.

The observed improvements in image quality can be attributed to underlying scattering physics. While Compton scattered data and attenuation measurements are both affected by the same underlying physical properties (density and photoelectric coefficient), the measurements are affected differently by these material properties. X-ray backscattering, which is a special case of Compton scattering, is known to highlight organic and low atomic number materials that have a low signature in transmission mode [40]. Thus, the addition of scattering data provides new cues that can be exploited in material recovery. As discussed above, another key feature of Compton scattering is that the broken raypaths add geometric paths through the scene that can be important in few-view imaging scenarios, leading to reduction in image artifacts.

In this paper, we focused on density reconstruction only, deferring photoelectric reconstruction to future work. However, the photoelectric coefficients can be recovered using a similar Landweber iteration. Because the Compton scattering effect (proportional to density) is significant at all energies (see Fig. 3), a reasonable density estimate is required for photoelectric recovery. The photoelectric profile could thus be recovered using a cyclic descent approach, in which Algorithm 1 above is used to estimate density, after which the estimated density is used to initialize the photolelectric recovery, and iteration between density and photoelectric reconstructions can be repeated as desired, yielding improved estimates on each iteration.

An important goal for future work is understanding the benefit of the proposed approach for a wider array of problems. Ideally future studies would include a wider variety of materials (textured materials, metal) as well as scenes with increased clutter. In addition, the geometric arrangement of X-ray sources and detectors could be optimized to ensure a more even coverage from all sides of the scanned object.

Multiple avenues exist to further develop X-ray systems that combine traditional attenuation-based imaging with Compton scatter data. Given the low count rates associated with Compton scattered data, larger area detectors or higher-flux sources would be desirable. Algorithmically, there are open questions on how best to combine transmission and scattering data during inversion; Fig. 10 and the related discussion explored this idea briefly. In part for computational reasons, we primarily used the energy-discriminating detectors to eliminate low-energy data where photoelectric effects are significant, and summed high-energy data together into a single energy bin. However, multiple high-energy bins could be used during reconstruction, and the optimal energy binning for detector data remains to be fully explored. This work explored only a single regularization strategy (Total Variation), so selection of alternate strategies and optimal parameter selection could improve performance. In addition, we explored only a single solution method (nonlinear Landweber) and future studies could explore potential advantages of algorithms such as ADMM, split-Bregman methods, or accelerated versions of steepest descent [34]. Finally, computational issues can be addressed by exploring the use of specialized hardware such as GPUs, and also exploring by geometries that would allow for analytic simplifications to image reconstruction.

## Appendix A

In this appendix, the computation of the differential $\partial _{\boldsymbol \rho } f\left ({\boldsymbol \rho,\boldsymbol p }\right)$ and the adjoint $\partial _{\boldsymbol \rho } f\left ({\boldsymbol \rho,\boldsymbol p }\right)^{T}$ operators of the Compton scattering model are described. For simplicity of notations and description, the adjoint and differential operators will be computed assuming one source and one detector only. Expanding the equations to several sources and detectors is straightforward. Starting with Eq. (6) and dropping the source and detector indices $i$ and $j$ and reordering some of the terms gives, for energy bin $m$,

\begin{align*} \boldsymbol {g}_{S}(m)=&\sum _{k}{} \sum _{l}{} I(E_{S_{k}})\Delta E_{S} S(\bar {\boldsymbol {r}}_{l,},\theta _{l},E_{S_{k}}) \\&\times \omega (l,k,m)\delta _{l}h(\boldsymbol {r}_{D'},\bar {\boldsymbol {r}}_{l},E'_{l,k}) \\&\times f(\bar {\boldsymbol {r}}_{l},\boldsymbol {r}_{S},E_{S_{k}})\rho (\bar {\boldsymbol {r}}_{l})\tag{17}\end{align*}

The photon counts due to $l^{th}$ interaction point and $k^{th}$ incident energy is given by:

\begin{align*}&\hspace{-1.2pc}\boldsymbol {g}_{M}(k,l) = I(E_{S_{k}})\Delta E_{S} S(\bar {\boldsymbol {r}}_{l,},\theta _{l},E_{S_{k}})\delta _{l} \\&\qquad \qquad \qquad \times h(\boldsymbol {r}_{D'},\bar {\boldsymbol {r}}_{l},E'_{l,k})f(\bar {\boldsymbol {r}}_{l},\boldsymbol {r}_{S},E_{S_{k}})\rho (\bar {\boldsymbol {r}}_{l})\tag{18}\end{align*}

which, using Eqs. (7) and (8), $\boldsymbol {g}_{M}(p)$, can be simplified as

\begin{align*}&\hspace{-1.2pc}\boldsymbol {g}_{M}(k,l) = I(E_{S_{k}})\Delta E_{S} S(\bar {\boldsymbol {r}}_{l,},\theta _{l},E_{S_{k}})\delta _{l}\Omega _{D'} \\&\qquad \qquad \times \exp \left ({-\boldsymbol {a}_{l}^{T} \boldsymbol \mu (E_{S_{k}})-\boldsymbol {b}_{l}^{T} \boldsymbol \mu (E'_{p})}\right)\rho (\bar {\boldsymbol {r}}_{l})\tag{19}\end{align*}

where only the last two terms depends on density. Eq. (17) can be computed using Eq. 18

\begin{equation*} \boldsymbol {g}_{S}(m) = \sum _{p}{}\omega (p,m)\boldsymbol {g}_{M}(p)\tag{20}\end{equation*}

which can be rewritten as a matrix multiplication, yielding a vector containing results for all energies:

\begin{equation*} \boldsymbol {g}_{S} =\boldsymbol {W}\boldsymbol {g}_{M}\tag{21}\end{equation*}

where the elements of matrix $\boldsymbol {W}$ are $\omega (p,m)$. The measured signal by the detectors $\boldsymbol {g}_{D'} $ given in Eq. (10) can be expressed in term of $\boldsymbol {g}_{M}$ as follow

\begin{equation*} \boldsymbol {g}_{D'} = \boldsymbol {S}\boldsymbol {W}\boldsymbol {g}_{M}\tag{22}\end{equation*}

Taking the derivative of Eq. (19) with respect to density gives

\begin{align*}&\hspace{-1.8pc}\delta \boldsymbol {g}_{M}(p) = I(E_{S_{k}})\Delta E_{S} S(\bar {\boldsymbol {r}}_{l,},\theta _{l},E_{S_{k}})\delta _{l}\Omega _{D'} \\&\qquad \qquad \times \exp \left ({-\boldsymbol {a}_{l}^{T} \boldsymbol \mu (E_{S_{k}})-\boldsymbol {b}_{l}^{T} \boldsymbol \mu (E'_{p})}\right)\boldsymbol {d}_{p}^{T}\delta \boldsymbol \rho\tag{23}\end{align*}

where

\begin{align*}&\hspace {-2pc}\boldsymbol {d}_{p}^{T}=-0.5 N_{A} \rho (\bar {\boldsymbol {r}}_{l}) f_{KN}(E_{S_{k}})\boldsymbol {a}_{l}^{T} \\&\qquad \qquad \qquad -0.5 N_{A} \rho (\bar {\boldsymbol {r}}_{l}) f_{KN}(E'_{p})\boldsymbol {b}_{l}^{T}+\boldsymbol {1}_{l}^{T}\tag{24}\end{align*}

where ${Z(\boldsymbol {r})}/{A(\boldsymbol {r})}$ is approximated by 0.5, a good approximation for most elements [22]. $\boldsymbol {1}_{l}^{T}$ is a row vector ordered lexicographically as in $\boldsymbol {a}_{l}^{T}$ and $\boldsymbol {b}_{l}^{T}$, which stores one for the element that corresponds to the interaction point and zeros otherwise. Eq. (23) can be simplified as

\begin{equation*} \delta \boldsymbol {g}_{M}(p) ={e}_{p}\boldsymbol {d}_{p}^{T}\delta \boldsymbol \rho\tag{25}\end{equation*}

where

\begin{align*}&\hspace {-2pc}{e}_{p} = I(E_{S_{k}})\Delta E_{S} S(\bar {\boldsymbol {r}}_{l,},\theta _{l},E_{S_{k}})\delta _{l}\Omega _{D'} \\&\qquad \qquad \qquad \times \, \exp \left ({-\boldsymbol {a}_{l}^{T} \boldsymbol \mu (E_{S_{k}})-\boldsymbol {b}_{l}^{T} \boldsymbol \mu (E'_{p})}\right)\tag{26}\end{align*}

The vector $\delta \boldsymbol {g}_{M}$ can be expressed as

\begin{equation*} \delta \boldsymbol {g}_{M} =\left [{\begin{array}{c} {e}_{1}\boldsymbol {d}_{1}^{T} \\ {e}_{2}\boldsymbol {d}_{2}^{T} \\. \\. \\. \\ {e}_{N_{\mathrm{ P}}}\boldsymbol {d}_{N_{\mathrm{ P}}}^{T} \end{array}}\right]\delta \boldsymbol \rho =\boldsymbol {M}\delta \boldsymbol \rho\tag{27}\end{equation*}

where $N_{\mathrm{ P}}=N_{E}N_{I}$, $N_{E}$ is the number of incident energies and $N_{I}$ is the number of interaction points. Using Eq. (22) and Eq. 27 gives the definition of $\partial _{\boldsymbol \rho }f\left ({\boldsymbol \rho,\boldsymbol p }\right)$

\begin{equation*} \delta \boldsymbol {g}_{D'}=\boldsymbol {S}\boldsymbol {W}\delta \boldsymbol {g}_{M}=\boldsymbol {S}\boldsymbol {W}\boldsymbol {M}\delta \boldsymbol \rho = \partial _{\boldsymbol \rho } f\left ({\boldsymbol \rho,\boldsymbol p }\right)\delta \boldsymbol \rho\tag{28}\end{equation*}

The transposed operator is then computed as

\begin{equation*} \partial _{\boldsymbol \rho } f\left ({\boldsymbol \rho,\boldsymbol p }\right)^{T}=\boldsymbol {M}^{T}\boldsymbol {W}^{T}\boldsymbol {S}^{T}\tag{29}\end{equation*}

Computing $\partial _{\boldsymbol p} f\left ({\boldsymbol \rho,\boldsymbol p }\right)$ and $\partial _{\boldsymbol p} f\left ({\boldsymbol \rho,\boldsymbol p }\right)^{T}$ follows similar steps as above.

## Appendix B

In this appendix, we briefly review the sinogram processing for transmission data. Our approach is a multi-energy extension of the dual-energy sinogram decomposition method presented by Ying *et al.* [44]. For more details, the reader is referred to [9]. Based on the relation in Eq.13, we can recover $c_{\tilde {\rho },i}$ and $c_{p,i}$ by minimizing the squared error between the measured projections and modeled projections. Here we define $\theta ^{i}=\left [{c_{\tilde {\rho },i},c_{p,i}}\right]$ as the unknown density and photoelectric projections along raypath $i$. Then, the log-normalized modeled projection along raypath $i$ for source energy $E_{s}$ is

\begin{align*}&\hspace{-1.8pc}K(\theta ^{i}) = -\ln \big [\int I(E_{s})exp(-c_{\tilde {\rho },i}f_{KN}(E_{s}) \\&\qquad \qquad \qquad -c_{p,i} f_{p}(E_{s}))dE_{s}] + \ln \int I(E_{S})dE_{S}\quad \tag{30}\end{align*}

Ying *et al.* studied the dual-energy case where the object is scanned twice with low- and high-energy source spectra. They used low- and high- energy measured sinograms to estimate sinograms in Compton (related to scaled density) and photoelectric space by minimizing the squared error between the summed low- and high-energy scans projection and a modeled project computed using Eq. 30, subject to non-negativity constraints. We generalize their result to multi-energies by seeking a solution that minimizes

\begin{equation*} (\theta ^{i}) = \mathop {\arg \min }\limits _{\theta ^{i}} ~({\boldsymbol {K}(\theta ^{i})}-\boldsymbol {m^{i}})^{T}\boldsymbol {\Sigma }({\boldsymbol {K}(\theta ^{i})}-\boldsymbol {m^{i}}). \tag{31}\end{equation*}

with nonnegativity constraints:

\begin{equation*} c_{\tilde {\rho },i}\geq 0 \quad \textrm {and} \quad c_{p,i}^{i}\geq 0\end{equation*}

In the equation above, vector $\boldsymbol {K}(\theta ^{i})$ is the $N_{E}$ dimensional column vector of modeled log-normalized mean values computed for different values of $E_{s}$, using Eq. 30 and $\boldsymbol {m^{i}}$ is the $N_{E}$ dimensional column vector of log-normalized measured projections. $\boldsymbol {\Sigma }=\text {diag}\{\boldsymbol {w}\}$ and $\boldsymbol {w}$ is the number of photon counts received in each energy bin. The $\boldsymbol {\Sigma }$ matrix acts as a weighting matrix, making the problem a weighted least squares problem, and was derived by Bouman and Sauer by finding a quadratic approximation of the Poisson log-likelihood function for X-ray transmission problem using a Taylor series expansion, which can be described as [45]–[47]. This weighting matrix $\boldsymbol {\Sigma }$ gives more weight to the raypaths and energy bins whose measured photon count is larger. In practice, for rays passing through objects with high attenuation, this gives more weighting to the high energy bins. In the case where only two energies are present and $\boldsymbol {\Sigma }$ is the identity, Eq. 31 reduces to the result in [44]. For the interested reader, [9] explores cases where multi-energy sinogram decomposition has advantages over dual-energy sinogram decomposition.
